# Cub Survival in a Wild Leopard (*Panthera pardus fusca*) Population

**DOI:** 10.3390/ani14182742

**Published:** 2024-09-23

**Authors:** Reuven Yosef, Swapnil Kumbhojkar, Jakub Z. Kosicki

**Affiliations:** 1 Eilat Campus, Ben Gurion University of the Negev, P.O. Box 272, Eilat 8810206, Israel; 2 Jhalana Wildlife Research Foundation (JWRF), GharkulSociety, Ganeshmala, Sinhagad Road, Pune 411030, India; swapnil.kumbhojkar@gmail.com; 3 Department of Avian Biology and Ecology, Adam Mickiewicz University, Uniwersytetu Poznanskiego, Str. 6, 61-614 Poznań, Poland; jzkosicki@gmail.com

**Keywords:** *Panthera pardus fusca*, Jhalana Forest Reserve, parturition, matriline, breeding success

## Abstract

**Simple Summary:**

We studied the survival of young Indian leopard cubs in the Jhalana Reserve Forest, India, during their first two years of life. Using data from trail cameras collected over four years (2018–2021), we calculated survival rates with a statistical method to identify when cubs are most at risk. All adults and cubs included in this study were identified at the individual level based on the rosette patterns on their flanks and their facial markings. We found that during the first year, the survival rate of the cubs was about 74%, indicating that this period is particularly challenging for their survival. In the second year, the survival rate improved to around 83%, showing that the cubs are more likely to survive as they grow older. Overall, 61% of the cubs survived in our population to become independent after two years of parental care. These findings are important for creating effective conservation strategies to protect leopard cubs in fragmented habitats.

**Abstract:**

We investigated the survival of cubs in a wild Indian leopard (*Panthera pardus fusca*) population in the Jhalana Reserve Forest (JRF), India. The research focuses on analyzing the survival of leopard cubs during their first two years of life. Survival functions were estimated using the Kaplan–Meier method based on data collected with trail cameras over four years from 2018 to 2021. We found that the mean survival probability of cubs during the first year of life was 0.739, indicating that this period is particularly challenging for their survival. In the second year, the survival probability increased to 0.831, reflecting an improvement in survival as the cubs grew older. The combined survival rate over the two-year period, calculated as the product of the first- and second-year survival rates, was 0.618. These findings highlight the critical periods in the early life stages of leopard cubs, which are essential for developing effective conservation strategies in fragmented habitats to enhance their survival.

## 1. Introduction

Leopards (*Panthera pardus*) are among the most versatile and widely distributed wild cats. They can adapt to almost every habitat, from human-inhabited urban areas to dense jungles and from lush agricultural fields to deserts and mountains [1]. However, owing to regional declines as a result of habitat fragmentation and degradation and human–wildlife conflicts [2], the leopard was recently assessed for the IUCN Red List of Threatened Species and has been listed as vulnerable since 2022 [3]. The global estimate of range reduction is 61% in just the past two decades [3]. In Africa, 37% of their historical natural habitats have been destroyed, and relatively large numbers of leopards have been killed by people either through trophy hunting or retaliatory killings due to actual or perceived threats to livestock [4]. However, the estimates of population changes in Asia are sporadic to non-existent. This is further complicated by taxonomic uncertainties that have yet to be resolved [5]. Most countries where the species has become extinct are in the Middle East and Southeast Asia (Hong Kong, Singapore, Israel, Jordan, Syria, Saudi Arabia, Libya, and Mauritania [6,7]).

The Indian leopard (*P. p. fusca*), prevalent beyond protected areas, is highly vulnerable to illegal wildlife trade (skins, bones, and other parts for use in traditional oriental medicine), especially among young individuals [8]. A regional survey revealed a rate of four leopards poached per week in India for the illegal wildlife trade [9]. Furthermore, it was reported that the illegal trade of leopard parts in Asia was on par with that of tigers (*P. tigris*) [10]. Indeed, in India, since 2000, an average of 3.5 young leopard seizure cases per month have been reported [3]. 

Leopard cubs face unique biological and ecological challenges that include predation by larger carnivores, infanticide by males who are not their sires, diseases, accidents, and starvation due to unsuccessful hunts by their mothers [11]. However, their most significant threats come from conflicts with humans, including illegal trade, poaching, and retaliatory killings [12,13]. The challenges in India are not well documented, and published studies are not necessarily based on field data [14]. These challenges underscore the vulnerability of leopard cubs and the urgent need to identify their survival probabilities in the wild.

Little is known about the breeding cycle of leopards in the wild [15]. Captive data show that females come into estrus at any time of the year and remain in heat for up to two weeks; young are born after a gestation period of 96 days. Mating in the wild lasts a day or two, and litters average two (range one to three) cubs. Young remain in the birthing den for the first two to three months, even if the mother is absent while foraging for prey, and accompany their mother when they are about three months old. The young are usually independent by 12–18 months, but dispersal varies from 15 to 36 months [15]. 

Leopard mothers play a crucial role in the survival of their cubs. They invest a significant amount of time and energy in raising their offspring, providing protection and guidance, and teaching them essential hunting and survival skills during their formative years [16]. As leopard cubs mature, they undergo a dispersal phase, leaving their mother’s territory to establish their home ranges. This period is fraught with risks, as they may encounter hostile territory, competition from resident leopards, and potential conflicts with humans [17]. It should also be noted that cub survival rates in leopards, like many other big cat species, can vary based on various factors such as habitat quality, availability of prey, competition, as well as human disturbances [2].

Despite some achievements in understanding the reasons for the decline in the number of this species still, the stage in the life history of a particular individual that is most crucial for their survival has yet to be determined. Therefore, conducting analyses of offspring survival from leaving the breeding ground to when they become independent is one key element in determining critical periods in the lives of individuals. It is essential because even protected areas do not ensure the undisturbed functioning of the population of this species. Because the species is long-lived, it seems necessary to estimate which moment in the life of young leopards is crucial for their survival.

The aims of this study are (1) to describe the juvenile survival of the Indian leopard during the first and second years of life and (2) to establish the critical period that influences survival.

## 2. Methods

### 2.1. Study Area

**Figure 1:**
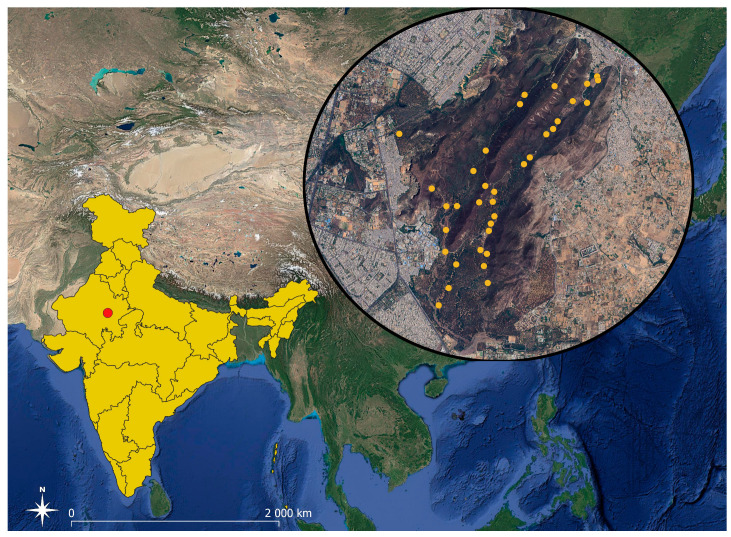
Location of Jhalana Reserve Forest in India (red circle). The locations of the camera traps are marked on the Google Maps background (yellow circles).

We conducted our study in the Jhalana Reserve Forest (JRF; 26°51′ N, 75°49′ E) located on the southeastern outskirts of Jaipur, the capital city of Rajasthan, India (Figure 1). Covering about 29 km^2^, JRF sits at an altitude of 516 m above sea level and falls under the category of Northern Tropical Dry deciduous forest. The landscape is dominated by low, flat-topped hills in the northern region, with deep erosion and dissected features [18]. Wildlife in JRF faces challenges due to the lack of natural water sources during dry months, relying on artificial waterholes created by the Rajasthan Forest Department. Notably, it boasts a relatively high leopard density (8.6 leopards/100 km^2^; Ref. [19]. Unlike many reserves, JRF lacks buffer zones and is entirely surrounded by urban and rural villages, making it a forest island. Ecotourism is practiced in JRF, with jeep tours conducted daily, while villagers collect wood and fodder in controlled areas without noticeable impact on the reserve’s predators [18]. 

### 2.2. Camera Traps

**Figure 2:**
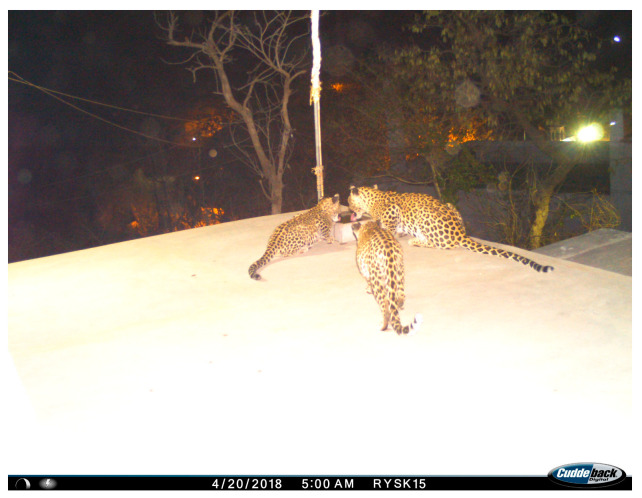
Sample photo from a camera trap. LK with her two cubs at a waterhole built by the locals on the roof of a local temple, Bhomiyaji Village (27°00′16″ N, 75°50′19″ E).

Camera traps were strategically placed along trails and near waterholes (Figure 2), positioned at a height of 45–50 cm above the ground. In fringe areas, the camera traps were enclosed in secure boxes attached to iron poles for extra safety measures. We deployed 18 trail cameras equipped with motion sensors (Cuddeback X-Change Color Model 1279, De Pere, WI, USA) to capture the activity of animals in JRF [19,20]. No baits or lures were used during this study [21].

### 2.3. Data Collection

For our analysis, we utilized data collected from 2018 to 2021 obtained through camera traps consistently positioned in the same locations throughout this period. Any breaks in monitoring were minimal, lasting only briefly when we replaced batteries. We took care not to disturb females in their dens, allowing us to capture their movements and the number of offspring they had. Therefore, in our study, the term “litters” refers to the number of offspring that accompanied the female after leaving the dens, indicating when a female was first seen with her most recent litter. It is worth noting that we may have missed cubs that were stillborn or died in the birthing dens before they emerged with their mothers and were photographed by the trail cameras. All individuals were identified by their unique facial markings and body patterning of the rosettes [19,20].

### 2.4. Statistical Analysis

**Figure 3:**
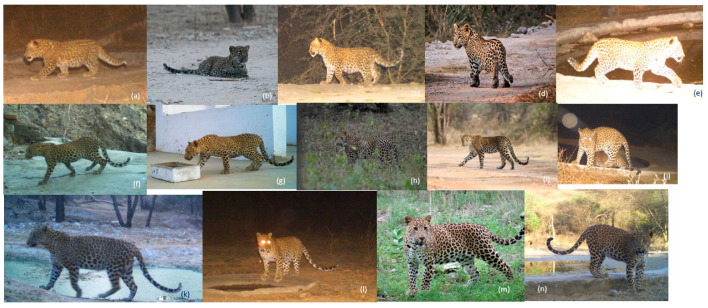
Photos of Indian leopard (*Panthera pardus fusca*) cubs in Jhalana Reserve Forest, showing their physical growth over the first 12 months of age. Age at (**a**,**b**) 1 month (13 February 2020, 16 March 2019), (**c**) 1.5 months (23 February 2020), (**d**) 2 months (23 May 2021), (**e**) 3 months (3 April 2020), (**f**) 4 months (16 June 2020), (**g**) 5 months (26 April 2018), (**h**) 6 months (16 October 2016), (**i**) 7 months (24 January 2018), (**j**) 8 months (4 January 2020), (**k**) 9 months (23 March 2020), (**l**) 10 months (17 March 2020), (**m**) 11 months (17 June 2022), and (**n**) 12 months (24 April 2021). Aging is based on field experience, observations, and dates from the camera traps. Photo credits: (**a**,**c**,**e**–**g**,**i**–**n**) Swapnil Kumbhojkar, JWRF & Rajasthan Forest Department (**b**,**h**) Mr. Abhinav Mudgal; (**d**) Surendra Chouhan Singh.

We calculated leopard cub survival by tracking changes in the number of young individuals following their mother. If fewer cubs were observed in subsequent camera trap photos compared to the family’s initial photo, we assumed that the individual had died midway between the two photographs. We established day 1 as the start of each individual’s lifespan, corresponding to the day of life on the date of the first photo minus two months (the length of time spent in the den). The endpoint was set as day 548 (18 months old, when leopards typically separate from their mother) and extended up to day 730 (two years old) if they appeared in subsequent photos. Cub age in the first photo was determined based on field experience, assuming that females first appeared with their young at waterholes when they were two to three months old (Figure 3).

Survival analysis of cubs within litters was conducted using life tables [22], tracking each cub’s life history from den departure to separation from their mother, usually between 18 and 24 months old. Unlike traditional approaches that assess the proportion of surviving cubs, we treated survivability as a time-dependent function [22,23,24,25,26]. This enabled us to identify critical time points during the breeding season when cubs depended on maternal care. Survival time for each litter was estimated using the Kaplan–Meier method [22], while the hazard ratio, derived from life tables, helped identify critical periods indicating the likelihood of death within a specific age group [22,23,24,25,26]. Seasonal survival comparisons were conducted using a multiple-sample test, an extension of Gehan’s generalized Wilcoxon test [27,28,29]. The analysis was performed using the ‘survival’ library [30,31] in R 4.3.3 [32].

## 3. Results

### 3.1. Study Population

In this study, the leopard population is primarily influenced by Arti, her daughters, and her granddaughters. The family unit includes Arti, her three daughters, (Flora and LK, born in 2013), and Sharmili (born in 2015), along with their daughters (Jalebi (born to Flora in 2015) and Tim Tim (born to LK in 2016)). Another family grouping comprises Nathwali and her daughter Leela, who were born in 2016. Additionally, independent of these two families, we also observed another female named Mrs. Khan. The average (±SD) interbirth interval for the cubs of these females was 11.4 ± 2.2 months.

### 3.2. Cub Survival

Over a total of 41,312 trap hours, we collected 30,694 photos, averaging 0.74 photos per hour. Unclear pictures were excluded from the analysis (N = 123, 0.4%). Of the 3201 (10.4%) leopard photos captured, 1582 (5.2%) depicted females with cubs.

**Figure 4:**
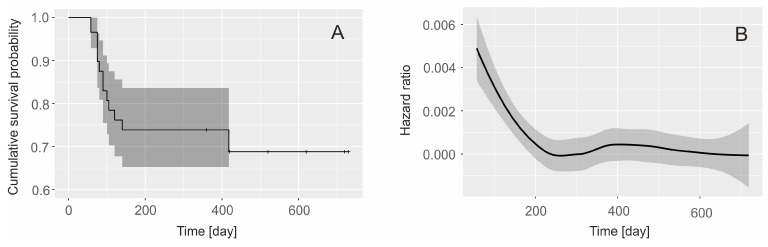
(**A**) The Kaplan–Meier survival functions for Indian leopard (*Panthera pardus fusca*) cubs; (**B**) hazard ratio probability of death during two years of a cub’s life.

Based on these photos, we monitored 16 litters born to nine females, resulting in a total of 33 cubs, of which 13 died (Table 1). The majority of these fatalities (N = 10 cubs, 76.9% of deaths) occurred within the first year of life, corresponding to a first-year survival rate of 0.739 (95% CI: 0.652–0.836) as estimated by the Kaplan–Meier method (Figure 4A). There were no significant differences in first-year survival rates between the four study years (chi-square = 4.3, df = 3, *p* = 0.2). The hazard ratio was highest shortly after the cubs left the dens and decreased steadily throughout the first year (Figure 4B).

In the second year, survival improved, with only two deaths (15.4% of total deaths), resulting in a second-year survival rate of 0.831 (95% CI: 0.768–0.999). This increase in survival between the first and second years was statistically significant (Gehan–Wilcoxon test, test value = 2.39, *p* < 0.02). Similar to the first year, no significant differences in second-year survival rates were found between the study years (chi-square = 5.3, df = 3, *p* = 0.5).

Overall, the combined survival rate of cubs from birth to independence at two years was 0.614 (95% CI: 0.526–0.793; see Figure 4A), with no significant variation across the study period (chi-square = 3.8, df = 3, *p* = 0.3).

## 4. Discussion

Our study highlights the critical period in the lives of Indian leopards, emphasizing the importance of the first 12 months. During this time frame, 30% of young individuals did not survive. Conversely, in the second year of life, the survival rate exceeded 83%, underscoring the significant impact of the female leopard’s reproductive success during her cub’s first year. While we did not observe infanticide by males during our study, previous reports have documented such behavior [11].

In South Africa, it was found that 47% of the African leopard cubs survived to independence: 55% survived the first three months, 44% survived to six months, 40% survived to nine months, 38% survived to 12 months, and 37% reached 18 months of age [33]. Another study reported combined annual survival rates for adults and subadults, showing slight differences between non-protected areas (0.55) and protected areas (0.88) in Southern Africa [34]. Snow leopards (*Panthera uncia*) in Mongolia, with minimal human disturbance, had a survival rate of 83% in the first two years of their lives [35]. A similar pattern was observed in cheetahs (*Acinonyx jubatus*), where cub survival on the Serengeti Plains was only 9.7%, compared to 45% in the Kgalagadi Transfrontier Park [36]. Predation by lions (*Panthera leo*), leopards, spotted hyenas (*Crocuta crocuta*), and Masai dogs (*Canis familiaris*) was a major factor in cheetah cub mortality. By contrast, in Asiatic Lions (*Panthera leo persica*) in western India, cub survival was 57% despite a 30% infanticide rate [37]. In pumas (*Puma concolor*), cub survival was closely tied to the fate of their mothers: 51% survived if the mother was alive, compared to only 14% if the mother died or was hunted [38]. These findings suggest that cub survival may vary greatly depending on individual circumstances within a specific population or family.

A crucial aspect of our analytical approach focused on determining when leopard cubs and their siblings achieve independence from their mothers. According to Owen et al. [17], African leopard cubs typically become independent around 11.6 months old, with a range of 11 to 13 months. In our study, the majority of young leopards left their mothers between 18 and 24 months of age. For instance, in April 2024, Barfi, a subadult female, separated from her mother, Jalebi, at 11 months old. However, we also observed that some Indian leopard cubs often stay with their mothers for up to two or three years, accompanying them within their home range [34].

It is important to note that when cubs vanish during their first year of life (not captured by photo traps or seen again), the reasons for this mortality remain unknown, as neither we nor the rangers of the Rajasthan Forest Department found any carcasses. However, it is plausible that females conceal the carcasses of their cubs, as described by [11], who observed the mother, Flora, hiding the carcass of a deceased cub in a cluster of thor cacti (*Euphorbia caducifolia*) to prevent striped hyenas (*Hyaena hyaena*) or jungle crows (*Corvus culminatus*) from accessing it.

Other studies on leopards have shown that female leopards increase their hunting efficiency, especially during the first year of their cubs’ lives, by targeting smaller prey more frequently [16]. However, our study stands out in this aspect due to the sedentary behavior of the females in Jhalana [19]. They primarily prey on stray dogs from the streets of neighboring Jaipur [20] and cattle carcasses from rural areas, ensuring a consistent food source across multiple years. Further, Jhalana boasts a relatively high leopard density (8.6 leopards/100 km^2^; [19]) compared to other regions in India (4.8/100 km^2^ in Maharashtra [35]; 7.96/100 km^2^ in northwest Bengal [39]) and globally (Nepal—1.5/100 km^2^ in the Terai region [40]; 3.31 and 3.45/100 km^2^ in Chitwan National Park [41]). Also, our study has unveiled intriguing aspects of Indian leopard behavior. The relatedness of females, abundant food resources, and minimal predation has fostered the emergence of alloparenting within our leopard community [42]. This unique phenomenon suggests a distinct scenario where the leopard population has adapted by exhibiting behaviors divergent from those of other wild populations to safeguard their cubs. This may directly contribute to the relatively high survival rate of the young and the low levels of predation observed among individual females in our study.

On the contrary, observations in this species have shown that females may cease parental care of current offspring to reproduce again [43]. Extended parental care does not necessarily impact the subsequent breeding attempt, thus not affecting the mothers’ overall fitness. Our photographs depict older cubs accompanying their mother and new litters, forming a sizable familial group, with the minimum age difference between litters being 12 months [19]. Additionally, [17] (2010) reported that females typically mate again when cubs are around ten months old, with an average interestrus period of 23 days and a gestation period of approximately 96 days, resulting in litters spaced approximately 14 months apart. Our data align with this to a large extent, but we observed some females having five litters in four years, suggesting that Indian leopards in JRF may have shorter interbirth intervals of 10–11 months. This is particularly noteworthy because it may be among the shortest interestrus periods observed (33 days [44] and 46 days in captivity [45]), potentially enabling faster recruitment of young under optimal environmental conditions in threatened populations [46]. This phenomenon warrants further investigation in other Indian and Asiatic populations to comprehend recruitment capabilities in wild populations and assess whether the unique island ecology of JRF has influenced leopard reproductive ecology and other behaviors.

Further, all future studies must take into account that while we present the survival rate of leopards during the first 24 months of life in JRF, this is only one part of their life history [33]. To determine if the population is stable or sustainable, these data must be combined with adult survival and reproduction rates [34,35]. This question can only be answered by considering all these factors together at the population level. Additionally, this is particularly important due to the unique urban–island biogeography of JRF.

## 5. Conclusions

In conclusion, this study elucidates the juvenile survival dynamics of Indian leopards within the Jhalana Reserve Forest, emphasizing the critical vulnerabilities encountered during the first two years of life. Our findings reveal that approximately 61% of cubs survive to achieve independence, underscoring the importance of maternal care and environmental factors in influencing survival rates. The identification of key periods of mortality risk provides a framework for developing targeted conservation strategies aimed at enhancing cub survivorship in fragmented habitats. Furthermore, the interplay between ecological conditions and anthropogenic pressures necessitates ongoing monitoring and adaptive management practices to mitigate threats to leopard populations. By integrating these insights into conservation planning, we can promote the long-term sustainability of Indian leopards and foster coexistence with human communities, thereby ensuring the preservation of this ecologically significant species within its natural habitat.

## Figures and Tables

**Table 1:** The table displays the number of young in a litter for each female in particular years (2018–2021). Litter ID represents the sequential number of the litter for each female. The color red indicates the first litter, blue the second, and green the third.

Female	2018			2019			2020			2021		
	Litter ID	Total Cubs	Dead Cubs	Litter ID	Total Cubs	Dead Cubs	Litter ID	Total Cubs	Dead Cubs	Litter ID	Total Cubs	Dead Cubs
Arti	1	2	0	1	2	0						
Flora				1	3	1	1	2	1	2	2	1
Jalebi	1	2	1	2	1	0	2	1	0			
Leela				1	2	0	1	2	0			
LK Female				1	2	2	2	2	2	3	3	0
Mrs Khan	1 *	1 *		2	3	1	2	2	0			
Nathwali	1	2	0	1	2	0	2	3	0	2	3	0
Sharmili				1	2	1	1	1	1	2	2	2
Tim Tim	1	2	2	2	1	0	2	1	0			

* unknown age, not included in analysis.

## Data Availability

All data are included in Table 1 in the manuscript. Additional data will be supplied upon reasonable request from the corresponding author.
